# Arthroscopy for the Painful Shoulder Arthroplasty: Indications, Outcomes, and Technical Considerations

**DOI:** 10.1007/s12178-026-10052-9

**Published:** 2026-07-27

**Authors:** Garrett R. Jackson, Sean Koetting, Justin T. Childers, Keith Kenter, Matthew J. Smith

**Affiliations:** 1 Department of Orthopaedic Surgery, University of Missouri, 1100 Virginia Avenue, Columbia, MO 65201 USA; 2 Department of Orthopedic Surgery, Orlando Health Jewett Orthopedic Institute, Orlando, FL USA

**Keywords:** Shoulder arthroplasty, Shoulder arthroscopy, Painful shoulder arthroplasty, Reverse shoulder arthroplasty, Total shoulder arthroplasty

## Abstract

**Purpose of Review:**

Persistent pain, stiffness, or suspected infection following shoulder arthroplasty remains a challenging clinical problem. Arthroscopy has emerged as a minimally invasive diagnostic and therapeutic tool in selected patients with painful anatomic total shoulder arthroplasty, reverse shoulder arthroplasty, or hemiarthroplasty. This review summarizes the current evidence regarding indications, outcomes, complications, and technical considerations for arthroscopy in the setting of pain following prior shoulder arthroplasty.

**Recent Findings:**

Shoulder arthroscopy after arthroplasty has been described for glenoid component loosening, polyethylene wear, rotator cuff pathology, biceps tendon disease, postoperative stiffness, impingement, and suspected periprosthetic joint infection. Reported outcomes suggest that arthroscopy may improve pain and function in selected patients, particularly after glenoid component removal, biceps procedures, capsular release, and lysis of adhesions. Arthroscopic tissue biopsy also demonstrates greater diagnostic utility than aspiration or serum inflammatory markers for suspected periprosthetic shoulder infection. Technical strategies, including initial subacromial evaluation and controlled glenohumeral entry through the rotator interval, may reduce iatrogenic implant damage during arthroscopic evaluation.

**Summary:**

Arthroscopy can be both diagnostic and therapeutic in well-selected patients with painful or stiff shoulder arthroplasty. Although it is not a substitute for revision surgery in cases of clear implant failure, instability, or massive cuff insufficiency, it may help clarify the diagnosis, treat focal pathology, and delay or avoid open revision in selected patients.

**Supplementary Information:**

The online version contains supplementary material available at 10.1007/s12178-026-10052-9.

## Introduction

Total shoulder arthroplasty (TSA) reliably relieves pain and restores function in most patients, but a persistent painful shoulder after implant remains a challenging problem [1–6]. Common causes of post-arthroplasty pain include implant loosening, periprosthetic joint infection, rotator cuff failure, component malposition or polyethylene wear, instability or subluxation, soft-tissue impingement, stiff shoulder with capsular contracture, long-head biceps tendon pathology, and glenoid bone loss [7–16]. Many such issues can be challenging to diagnose solely through imaging. Arthroscopy has emerged as both a diagnostic and therapeutic tool in the management of the painful post-arthroplasty shoulder [10, 17–21]. Although primarily described as a diagnostic adjunct through obtaining cultures or visualizing component fixation, several studies have shown that arthroscopy can improve pain and function when used to release capsular adhesions or through removal of loose glenoid components [22–24]. In a recent study of 19 patients with noninfectious stiffness and anterior shoulder pain following reverse shoulder arthroplasty (RSA), Ardebol et al. [25] reported that arthroscopic lysis of adhesions and subcoracoid decompression significantly improved pain in 74% of patients, range of motion in 79%, 68% returned to activities and 74% of patients were satisfied. No postoperative complications were reported. Similarly, studies have found that patients who underwent arthroscopic glenoid component removal reported significant improvement in patient-reported outcomes measures (PROMs) and pain, with a low rate of open revision surgery [22]. Overall, arthroscopy following shoulder arthroplasty is generally low-risk and can obviate open revision in select patients.

Indications for shoulder arthroscopy after arthroplasty continue to expand to include unexplained pain or stiffness, suspected component loosening, evaluation of rotator cuff or biceps pathology, scope biopsy for infection, capsular release for treatment of adhesive capsulitis or impingement [11, 19, 24]. Understanding the indications and outcomes of patients who underwent shoulder arthroscopy following shoulder arthroplasty is essential for optimizing patient outcomes and satisfaction. Thus, this review aims to provide an updated overview of the indications, outcomes, and complications following shoulder arthroscopy in the painful shoulder arthroplasty patient.

## Glenoid Component Loosening and Polyethylene Wear

**Fig. 1:**
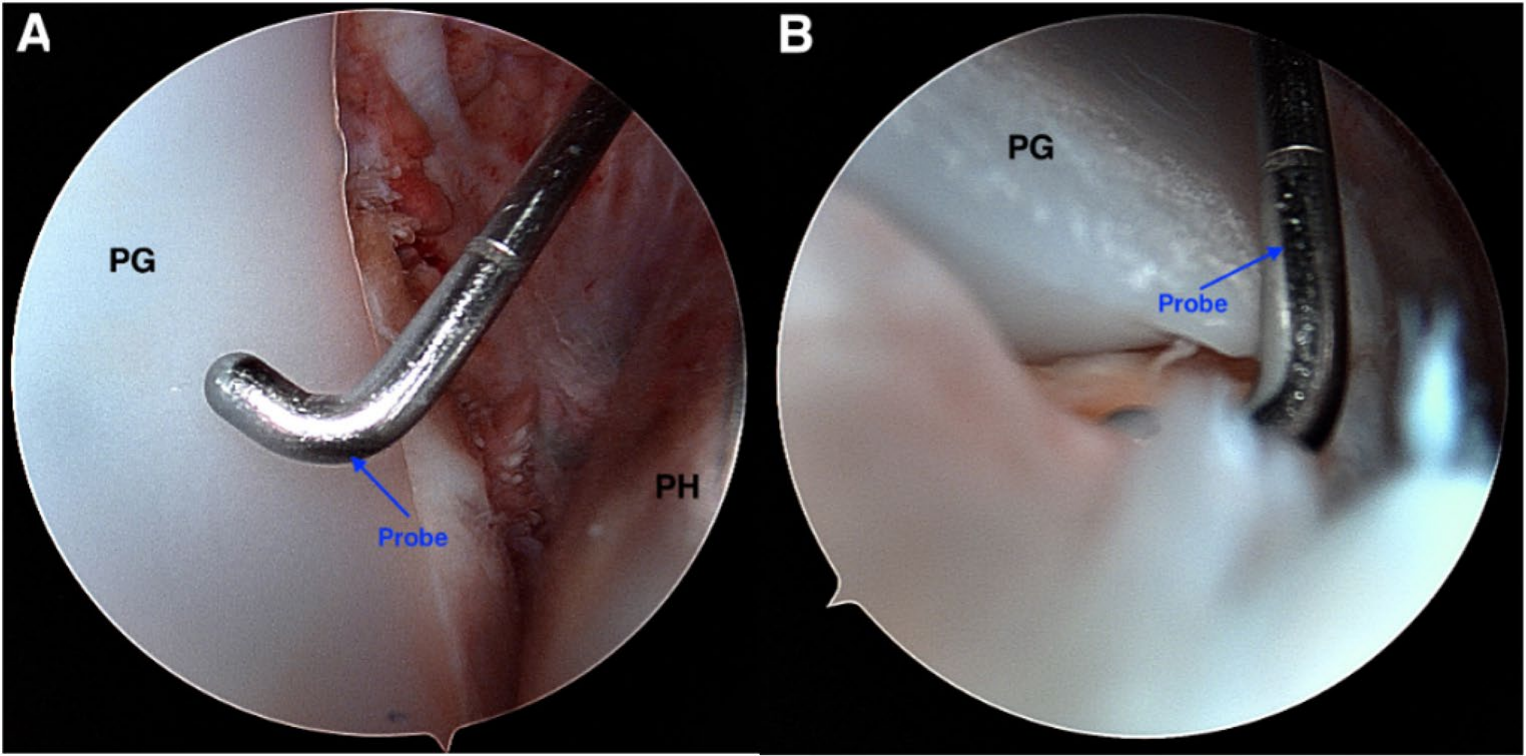
(**A**) Arthroscopic image of a right shoulder looking from the posterior portal into the glenohumeral joint with a stable glenoid. (**B**) Arthroscopic image of a right shoulder looking from the posterior portal into the glenohumeral joint with a loose glenoid. Legend: PH, prosthetic humeral head; PG, prosthetic glenoid

Aseptic loosening of the glenoid component is a leading cause of pain and failure after shoulder arthroplasty [14, 26]. Standard evaluation includes radiographs/CT and infection workup; however, if studies are equivocal, diagnostic arthroscopy can directly visualize the glenoid–implant interface **(**Fig. 1**)**, and the polyethylene glenoid component can be removed with minimal soft-tissue disruption [24]. Several studies have shown that arthroscopic glenoid removal, often with bone grafting of the glenoid vault, can relieve pain and improve range of motion in low-demand patients [22, 27, 28]. For example, in a systematic review of six studies including 25 shoulders that underwent arthroscopic glenoid component removal, Ozdag et al. [22] reported that all patients had significant improvement in PROMs. Changes in range of motion were variable. Forward elevation was documented in 16 patients preoperatively and in 17 patients postoperatively, with a mean of 117.5° and 117.4°, respectively. External rotation was reported in 16 patients, which demonstrated a preoperative mean of 32.5° and a postoperative mean of 34.7°. Despite the improvements in PROMs and external rotation, 3 patients did experience postoperative complications, including later revision surgery due to continued pain (*n* = 2/25) and an exacerbation of lymphedema (*n* = 1/25).

### Rotator Cuff Pathology

**Fig. 2:**
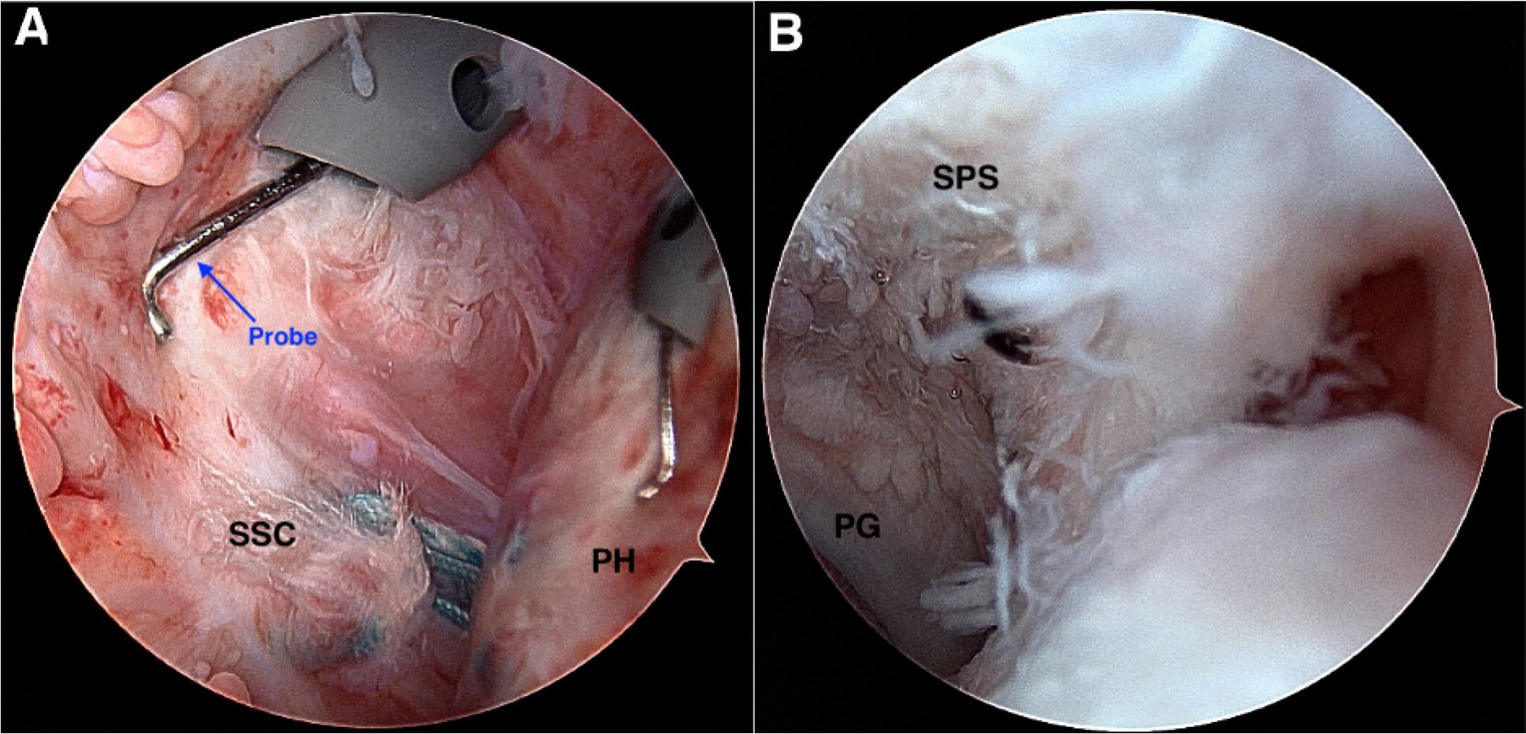
(**A**) Arthroscopic image of a right shoulder looking from the posterior portal, visualizing subscapularis insufficiency. (**B**) Arthroscopic image of a right shoulder looking from the lateral portal, visualizing a full-thickness supraspinatus tear. Legend: PG, prosthetic glenoid; SSC, subscapularis tendon; SPS, supraspinatus tendon

Rotator cuff insufficiency after anatomic TSA (aTSA) can cause pain, weakness, and shoulder instability. While advanced imaging such as CT is widely accessible, fast, and excellent for evaluating bone structures, its use involves ionizing radiation and is prone to imaging artifacts from metallic implants, though newer metal artifact reduction techniques and dual-energy CT have mitigated some of these issues [29, 30]. CT remains valuable for assessing implant loosening, fractures, heterotopic ossification, hardware status, and muscle quality, but its ability to detect subtle soft tissue pathology, can be limited. MRI, on the other hand, offers superior soft-tissue contrast without radiation exposure and allows for detailed multiplanar imaging, yet it poses challenges in the arthroplasty setting due to metal-induced artifacts that obscure visualization around implants [31]. Although strategies like using lower field strengths and fast-spin echo sequences can reduce these artifacts, MRI remains costly and time-consuming and may be contraindicated in patients with certain implants or severe claustrophobia [31–33]. In contrast to these imaging modalities, arthroscopy provides direct visualization of both soft tissue and implant interfaces, allowing surgeons to diagnose and address intra-articular pathology in real time without being hindered by metal artifacts. Arthroscopy allows for direct visualization of the rotator cuff without disrupting the implant **(**Fig. 2**)**. However, surgical repair of large post-arthroplasty cuff tears is challenging. In a series of 18 shoulders undergoing cuff repair after aTSA, Hattrup et al. found that only 4 patients had successful repair and most had persistent pain (mean visual analog pain score [VAS] = 5.6) and limited forward elevation (mean = 78 degrees), and external rotation (mean = 54 degrees) [34]. Given these poor outcomes, arthroscopic management of a post-aTSA cuff tear is usually limited to debridement and biceps tenotomy/tenodesis if indicated; large tears or complete cuff failure typically mandate conversion to a reverse prosthesis. However, arthroscopy can be used diagnostically to confirm the presence and size of a cuff tear, which may then guide revision strategy.

**Fig. 3:**
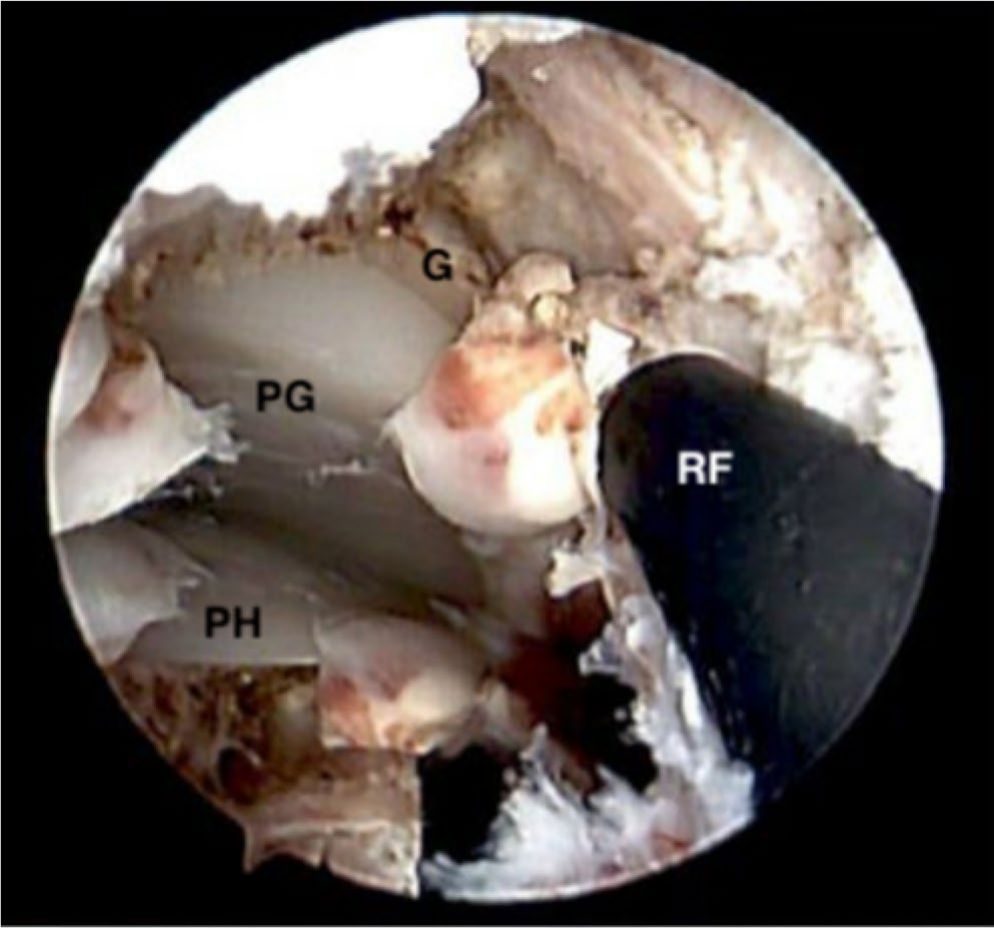
Arthroscopic image of a right shoulder looking from the anterolateral portal into the rotator interval. Legend: PH, prosthetic humeral head; PG, prosthetic glenoid; G, glenoid; RF, radiofrequency probe

The authors’ preferred technique when performing arthroscopy of a total shoulder begins with the introduction of the camera into the subacromial space via a standard posterior portal. Subacromial debridement is performed through an anterolateral portal, allowing thorough assessment of the bursal cuff (Fig. 3**)**. The arthroscope is then transitioned to the anterolateral portal, and an anterior portal is established. Utilizing the coracoid process as a guide, the rotator interval is opened, permitting safe entry into the glenohumeral joint from the anterior aspect. The authors favor this technique for its ability to minimize the risk of iatrogenic damage to prosthetic components while also facilitating rotator interval release in patients with postoperative stiffness.

### Long-Head Biceps Pathology

Long-head biceps tendonitis is an under-recognized source of anterior shoulder pain after arthroplasty. A series of patients with anterior shoulder pain and biceps tendonopathy after TSA (*n* = 5) or hemiarthroplasty (*n* = 3) showed that arthroscopic biceps tenodesis or debridement resulted in improvements in shoulder range of motion [35]. In the hemiarthroplasty group, average flexion increased by 36° (range, 68°-104°), external rotation improved by 23° (range, 11°–34°) and internal rotation reached at the level of L4. In comparison, the TSA group reported a mean increase in flexion of 50° (range, 66°–166°), external rotation by 27° (range, 22°–39°), and internal rotation to the L3 level. Every shoulder showed an increase in Hospital for Special Surgery (HSS) scores, and all patients were satisfied with their outcomes. Mean pain scores decreased significantly from 6.9 preoperatively (range, 4–9) to 1.4 postoperatively (range, 0.5–2) on a 10-point scale, where 10 represented the highest level of pain.

### Arthroscopic Capsular Release for Adhesive Capsulitis or the Stiff Shoulder Replacement

Postoperative shoulder stiffness after arthroplasty can limit patients’ motion and cause significant shoulder pain and debilitation. Arthroscopic capsular release and lysis of adhesions have been used in stiff, well-fixed implants. Guild et al. [18] reported arthroscopic debridement of adhesive capsulitis in three patients with stiff shoulders. At a mean follow-up of 18.6 months, patients had a mean postoperative VAS pain score of 2.5 from a preoperative score of 8.2. Additionally, Ardebol et al. [25] found that arthroscopic lysis of adhesions and release of the rotator interval in RSA patients with noninfectious stiffness significantly improved VAS (Δ-1.1), American Shoulder and Elbow Surgeons (Δ16.2), Subjective Shoulder Value (Δ18.5), FF (Δ23°), and external rotation (Δ15°). Additionally, in a study performed to analyze the outcomes of open and arthroscopic capsular release following TSA, Wagner et al. [21] analyzed 19 patients with a mean follow-up of 2.3 years. All 19 patients experienced persistent shoulder stiffness after undergoing aTSA with 5 requiring open capsular release and 14 undergoing arthroscopic capsular release. Improvements were reported in forward flexion (from 77° to 117°), abduction (49° to 98°), external rotation (9° to 19°), and internal rotation at 0° abduction (from the sacrum to L1), along with a significant decrease in pain scores (from 4.1 to 2.3). Despite these improvements, reoperation was required in 37% of patients (*n* = 7) following the index capsular release. The reoperation-free survival was 76% at 2 years and 53% at 5 years, while revision-free survival at both time points was 83%. Additionally, 16% of patients (*n* = 3) underwent a repeat capsular release. In total, complications were reported in 11 patients (58%), including postoperative stiffness (*n* = 9), infection (*n* = 1), subscapularis rupture (*n* = 2), glenoid component loosening (*n* = 3), and persistent pain with weakness necessitating reoperation (*n* = 1).

Overall, early arthroscopic release (within one year of arthroplasty) is recommended for post-arthroplasty stiffness, since prolonged adhesion reduces effectiveness [25]. Procedures may include anterior capsular release, inferior capsulotomy, and subacromial clearance. In many cases these patients also have concomitant synovitis or minor impingement that can be addressed concurrently.

### Arthroscopic Biopsy for Periprosthetic Joint Infection

Infection following arthroplasty can result in high morbidity, with an incidence ranging from 0.7 to 7% [36]. Periprosthetic shoulder arthroplasty infection diagnosis continues to be a challenge and it is debated as to whether or not the performance of a preoperative arthroscopic tissue culture yields superior outcomes when compared to a tissue biopsy obtained at the time of revision surgery [36]. In a recent systematic review of 75 aTSA patients, 60 RSA patients, and 64 hemiarthroplasty patients, arthroscopic tissue biopsy yielded positive cultures in 46.7% (*n* = 56/120) of samples compared to 40.8% (*n* = 64/157) of positive cultures obtained from open biopsy during revision surgery. Pooled results from the meta-analysis demonstrated that arthroscopic tissue cultures provided superior diagnostic accuracy for periprosthetic shoulder infection, with a sensitivity of 0.76 (95% CI, 0.57–0.88) and specificity of 0.91 (95% CI, 0.79–0.97). In comparison, joint aspiration yielded lower sensitivity (0.15; 95% CI, 0.03–0.48) but comparable specificity (0.93; 95% CI, 0.65–0.99), while elevated erythrocyte sedimentation rate or C-reactive protein levels showed similarly limited sensitivity (0.14; 95% CI, 0.02–0.62) and a specificity of 0.83 (95% CI, 0.56–0.95). The authors concluded that obtaining arthroscopic tissue cultures is comparable to the current gold standard of obtaining cultures intraoperatively during revision surgery but was superior to conventional techniques of joint aspiration and serum marker testing. Similarly, in a study of 19 patients who underwent arthroscopic biopsy to evaluate a painful shoulder arthroplasty for potential infection, Dilisio et al. [37] found that all arthroscopic biopsy results were consistent with cultures obtained during revision procedures, resulting in a 100% sensitivity, specificity, negative predictive value, and positive predictive value. Conversely, fluoroscopy-guided glenohumeral aspiration demonstrated a sensitivity of 16.7%, specificity of 100%, with a positive predictive value of 100% and a negative predictive value of 58.3%. These results highlight the reliability of arthroscopic tissue biopsy for diagnosing periprosthetic shoulder infection.

## Conclusion

Overall, in well-selected patients with painful shoulder arthroplasty, arthroscopy is safe and can be both diagnostic and therapeutic. Arthroscopy offers the distinct advantage of direct, real-time visualization of both soft tissue and implant interfaces, eliminating the diagnostic limitations imposed by metal artifact seen in CT and MRI. Unlike advanced imaging, arthroscopy allows simultaneous diagnosis and treatment, particularly in complex postoperative shoulders where imaging may be inconclusive. Additionally, it provides a minimally invasive way to debride synovitis, release adhesions, debride cuff edges, and remove loose glenoid components with high patient satisfaction and improved outcomes, with relatively low complication rates. Furthermore, arthroscopic tissue cultures have proven to be a useful tool to help guide the management of periprosthetic joint infections in shoulder arthroplasty. The surgeon should consider this technique in patients with a painful or dysfunctional shoulder replacement.

## Electronic Supplementary Material

Below is the link to the electronic supplementary material.

Supplementary Material 1

Supplementary Material 2

Supplementary Material 3

## Data Availability

No datasets were generated or analysed during the current study.

## Key References

- Ardebol J, Noble MB, Galasso LA, Hartzler RU, Werner BC, Millett PJ. Therapeutic arthroscopy for noninfectious stiffness and anterior shoulder pain after reverse shoulder arthroplasty leads to clinical improvement in most patients with a low complication rate. Journal of Shoulder and Elbow Surgery. 2025.
  - ○ This recent multicenter retrospective review that examines the utility of therapeautic arthroscopy for noninfectious stiffness and anterior shoulder pain following RSA at 1-year follow-up, reporting findings of improved clinical outcomes with a low rate of complications with greater functional improvement in earlier intervention.
- Ozdag Y, Baylor J, Hayes D, Grandizio LC. Arthroscopic Removal of the Polyethylene Glenoid Component After Total Shoulder Arthroplasty: A Systematic Review. Journal of Shoulder and Elbow Arthroplasty. 2022;6:247154922211429.
  - ○ This systematic review evaluates the arthroscopic removal of loose polyethylene glenoid components in a small total cohort (*n* = 25 shoulders) demonstrating improvements in both pain and PROMs, however, noting the necessity for further large-scale, prospective investigations.
- Tat J, Tat J, Faber K. Arthroscopic tissue biopsy as a preoperative diagnostic test for periprosthetic shoulder arthroplasty infections: a systematic review and meta-analysis. Journal of Shoulder and Elbow Surgery. 2023;32(7):1545–54.
  - ○ This recent systematic review and meta-analysis reports that arthroscopic tissue biopsy provides equivalent diagnostic accuracy compared to cultures obtained during revision arthroplasty, exhibiting superior sensitivity compared to traditional techniques such as joint aspiration and measuring serum inflammatory markers. This study underscores arthroscopy’s value in establishing a diagnosis and guiding staged versus single-stage revision shoulder arthroplasty.
- Ziegler R, Mashni SJ, Fleckenstein CM, Hasan SS. Arthroscopic Removal of Loose Glenoid Component in Anatomic Total Shoulder Arthroplasty. Arthroscopy Techniques. 2024;13(7).
  - ○ This recent technical note describes a reproducible arthroscopic method for evaluating and removing a loose glenoid component after aTSA, while minimizing disruption of the surrounding soft tissues and retained humeral component. It provides practical guidance for portal placement, implant protection, and glenoid management.
- Guild T, Kuhn G, Rivers M, Cheski R, Trenhaile S, Izquierdo R. The Role of Arthroscopy in Painful Shoulder Arthroplasty: Is Revision Always Necessary? Arthroscopy: The Journal of Arthroscopic & Related Surgery. 2020;36(6):1508–14.
  - ○ This retrospective study examines patients with postoperative pain following aTSA and rTSA and determines whetehr arthroscopy is a viable means of diagnosis and treatment. The study found that 46% of patients were able to be successfully treated with arthroscopy, thus avoiding the need for revision arthroplasty. Further, in patients requiring revision open cultures and frozen sections demonstrated complete concordance swith those obtained during arthroscopy, highlighting the diagnostic accuracy of arthroscopy.
