# Supplementary figures and images for: Arthroscopy for the Painful Shoulder Arthroplasty: Indications, Outcomes, and Technical Considerations
Source: Curr Rev Musculoskelet Med. 2026 Jul 27;19(1):59. doi: 10.1007/s12178-026-10052-9 (PMC13407823; doi:10.1007/s12178-026-10052-9)

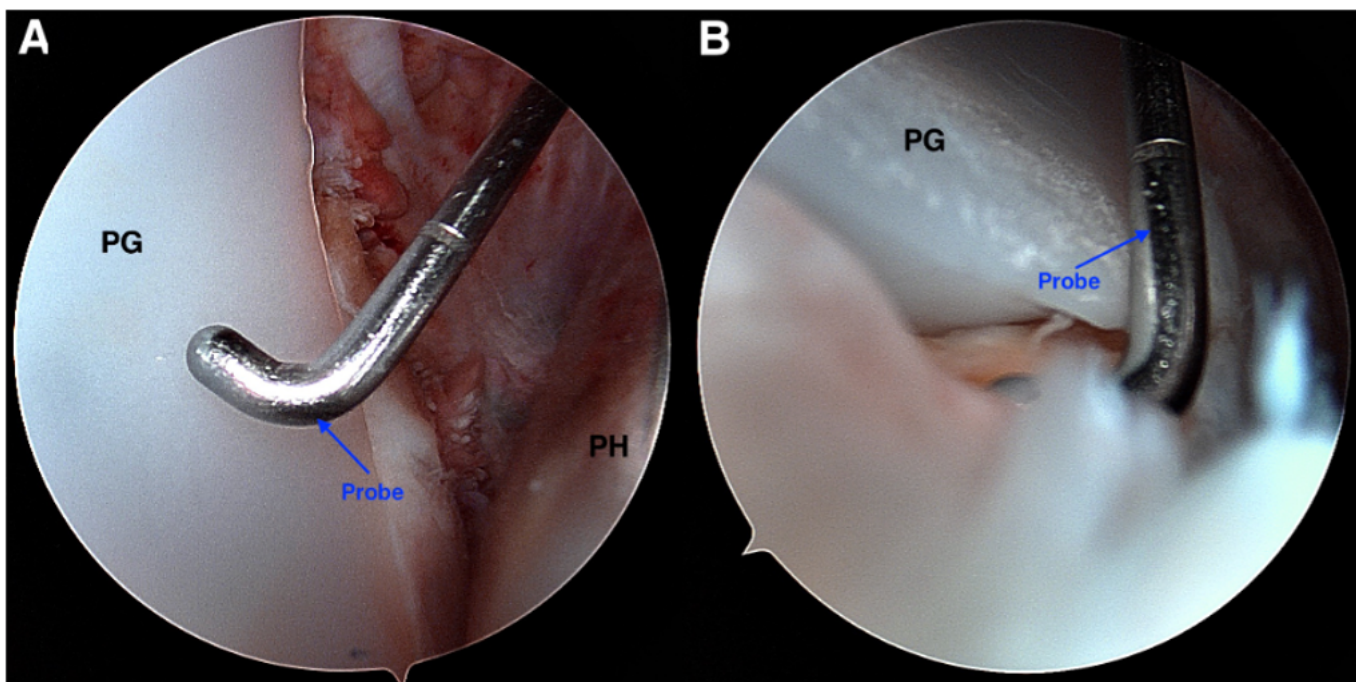

Supplement: Supplementary file 1 — Supplementary Material 1 [file 12178_2026_10052_MOESM1_ESM.pdf]

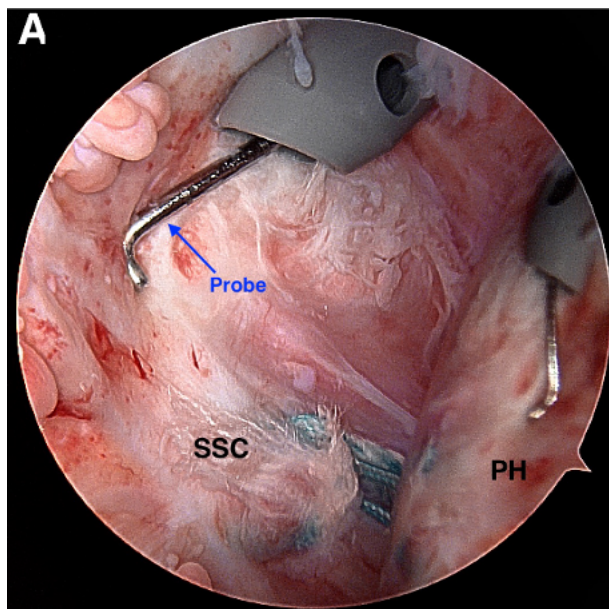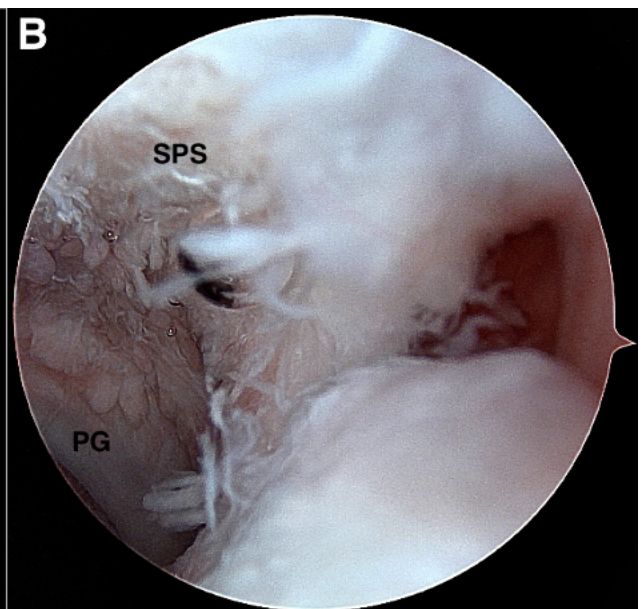

Supplement: Supplementary file 2 — Supplementary Material 2 [file 12178_2026_10052_MOESM2_ESM.pdf]

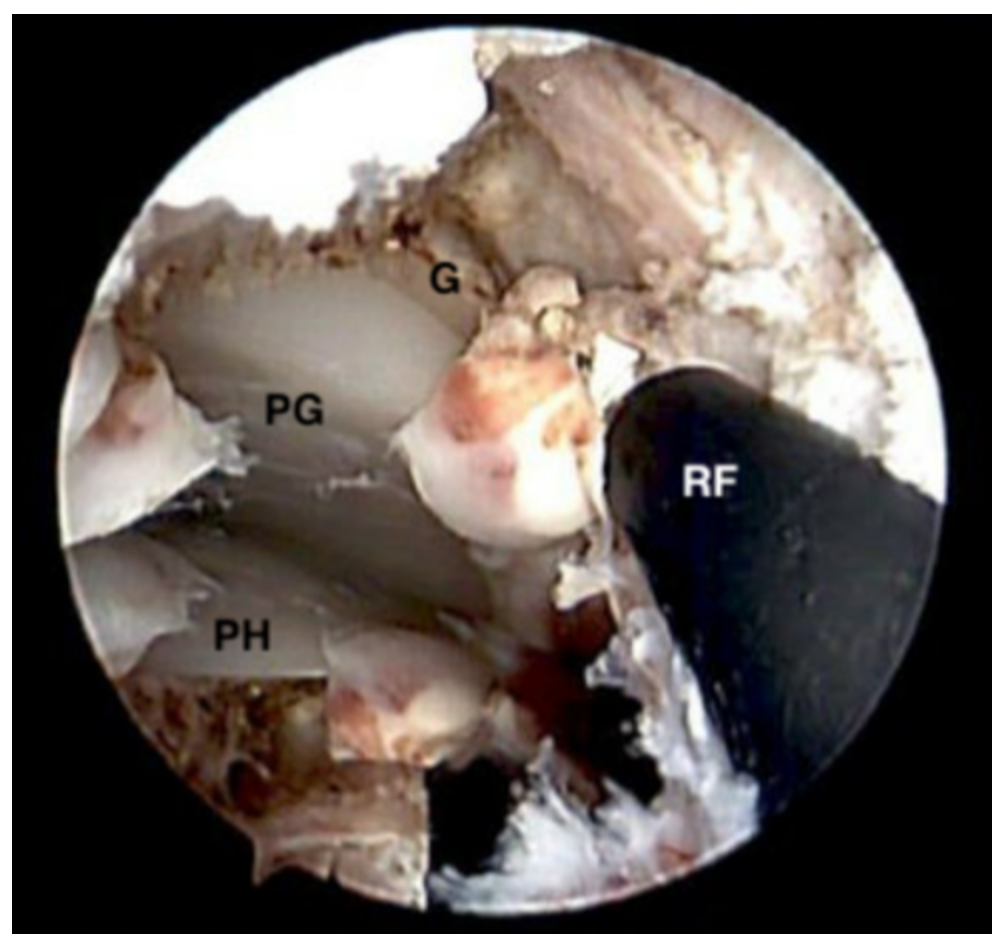

Supplement: Supplementary file 3 — Supplementary Material 3 [file 12178_2026_10052_MOESM3_ESM.pdf]
